# The cerebrospinal fluid biomarker profile in an HIV-infected subject with Alzheimer’s disease

**DOI:** 10.1186/s12981-015-0063-x

**Published:** 2015-07-14

**Authors:** Signar Mäkitalo, Åsa Mellgren, Ellen Borgh, Lena Kilander, Tobias Skillbäck, Henrik Zetterberg, Magnus Gisslén

**Affiliations:** Department of Infectious Diseases, Gävle Hospital, Gävle, Sweden; Clinic of Infectious Diseases, South Älvsborg Hospital, Borås, Sweden; Department of Infectious Diseases, University of Gothenburg, Sahlgrenska University Hospital, Gothenburg, Sweden; Department of Respiratory Medicine, University of Gothenburg, Sahlgrenska University Hospital, Gothenburg, Sweden; Department of Public Health and Caring Sciences/Geriatrics, Uppsala University, Uppsala, Sweden; Clinical Neurochemistry Laboratory, Institute of Neuroscience and Physiology, The Sahlgrenska Academy at the University of Gothenburg, Mölndal, Sweden; UCL Institute of Neurology, Queen Square, London, UK

**Keywords:** Alzheimer’s disease, HIV, CSF biomarkers

## Abstract

It is a challenge to differentiate between HIV-associated neurocognitive disorders (HAND) and other types of neurocognitive disease in the ageing HIV-infected population. Here we describe a 63 year old HIV-infected woman who had a history, neuropsychological test result, and PET examination consistent with characteristic Alzheimer’s disease (AD). The cerebrospinal fluid (CSF) biomarker profile was analogous to the profile typically found in AD in HIV-negative patients with increased t-tau and p-tau, a decreased level of Aβ42 and normal levels of CSF neurofilament light protein and sAPPα and sAPPβ, distinctly different from findings in HIV-associated dementia (HAD). Assessment of CSF biomarkers may be a valuable tool for clinicians to distinguish between HAD and AD.

## Background

Neurological complications were common features of HIV-infection before the era of effective antiretroviral treatment (ART) and more than 20% of HIV-infected patients without treatment developed HIV-associated dementia (HAD) in late disease [1]. Severe neurological complications are today rare in patients on suppressive ART [2] while milder forms of cognitive impairment are more frequently reported [3, 4]. Neurological complications have also been described in patients on ART with suppressed viral replication in plasma but detectable viral load in the CSF, so called symptomatic CSF escape [5, 6].

In HIV, a continuous immune activation of the central nervous system (CNS) is still present despite several years of otherwise effective ART [7, 8]. This chronic inflammation, together with a long lifespan of patients on ART, has raised concerns of whether HIV infection might interact with, or even potentiate, the development of other neurocognitive diseases such as AD. Older HIV-infected persons may also be more at risk of developing HAND than younger individuals [9]. In any event, age-related morbidity will be an increasing challenge in the aging HIV-infected population. The number of HIV-infected patients, who develops neurocognitive diseases, including AD, will increase with time and it will become a challenge in those cases to differentiate between AD and different forms of HAND.

The CSF biomarker profile has been extensively studied in AD and a typical pattern is increased CSF levels of total (t-tau) and hyperphosphorylated protein tau (p-tau), indicating cortical neurodegeneration and tangle pathology, and decreased levels of amyloid-β_1–42_ (Aβ42), as a sign of senile plaque pathology [10]. In HAD, a slight different pattern could be found with normal p-tau, normal or increased t-tau, and normal or decreased Aβ42 [11–16]. Other CSF markers, such as soluble amyloid precursor proteins (sAPPs) and neurofilament light protein (NFL) also have a different pattern in AD as compared to HAD, with reduced sAPP levels and increased NFL levels in HAD (these levels are typically normal in AD) [11].

The CSF biomarker profile in patients with concomitant disease has not yet been defined. Here we report CSF biomarkers in an HIV-infected patient with AD.

## Case presentation

The patient is a woman born in 1942 who was diagnosed with HIV 1998. She was infected sometimes between 1994 and 1996. ART was initiated 1998 when her CD4-cell count was 200 cells/μL. Plasma HIV RNA has been suppressed below 50 copies/μL since 2000, with only a few minor blips. CD4^+^ T cell count has remained between 800 and 1000 cells/μL for the last 8 years. The patient has a strong heredity of AD where her mother, two aunts on her mother’s side and her grandmother had AD with an onset between 70 and 80 years of age.

The patient’s cognitive impairment started in 2005 at the age of 63 years and since 2006 she has been an outpatient at the Memory Clinic of Uppsala University Hospital. In 2006, minimal mental state examination (MMSE) showed 29 points out of 30, and in where she failed to perform the simple figure copying. A CT scan of the brain in 2006 showed no signs of abnormality. A more extensive neuropsychological examination was done in 2007 and it showed predominantly normal results except for a very low performance in spatial short time memory and spatial episodic memory corresponding to right side posterior cortical functions. The conclusion was an overall good cognitive ability but with a selected cognitive impairment in spatial abilities corresponding to the patient’s own experiences. Repeated MMSE evaluation were initially stable, but a substantial performance decrease was revealed after 2011 (October 2006: 29, April 2009: 29, October 2009: 28, November 2010: 26, November 2011: 28, December 2012: 21, and October 2014: 18). In October 2009 a PET-FDG examination was done that showed decreased glucose uptake bilaterally in the apical parts of the parietal lobes, decreased uptake in the left anterior singulum and in the basal parts of the parietal lobes and the right posterior thalamus, all findings conclusive with neurodegeneration typical for AD with a frontal involvement. ApoE testing was not accomplished.

In November 2009, the patient described her problems as a change in temper, being nearer to tears, having less tolerance for stress, problems finding her way around and problems finding words. She started medication for AD with colinesterasinhibitor donepezil 5 mg qd in addition to her other prescribed medications: venlafaxine, rosuvastatin, cetirizin and ART (lamivudine, abacavir, lopinavir and ritonavir). In 2010, donepezil was changed to galantamine 8 mg qd due to side effects. After 2011, the patient had increased memory difficulties and decreased stress tolerance. She had difficulties to cook and was unable to bake. Her problems with orientation increased even at home. Her husband described her short-term memory as substantially decreased and he had to remind her of her medication. In 2012 the AD treatment with galantamine was increased to 24 mg qd. Raltegravir was temporarily added to her ART in October 2010 for a few months but was halted when CSF HIV RNA remained suppressed.

### CSF analyses

**Figure 1:**
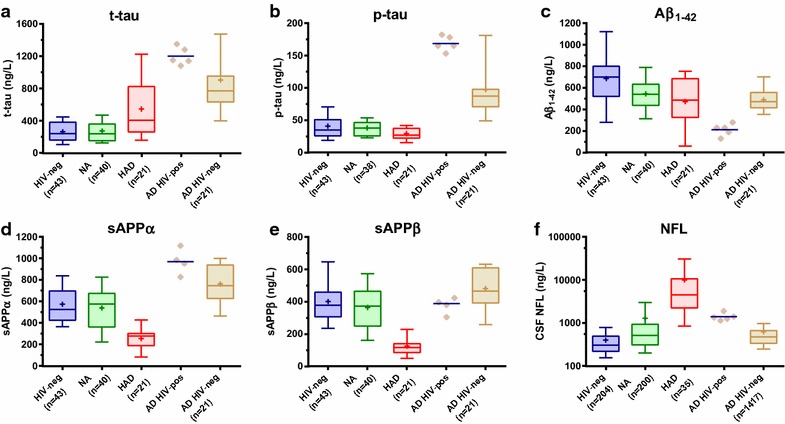
The concentrations of the CSF biomarkers analyzed in the reported case (AD HIV-pos) as compared to HIV-negative controls, neuroasymptomatic untreated HIV-infected (NA), HIV-associated dementia (HAD), and HIV-negative Alzheimer’s disease (AD) obtained from previous studies: **a**–**e** [11]; AD in **f** [19], other groups [11]. *Boxes* encompass interquartile ranges (IQR) with median (*line*) and mean (“ + ”), while whiskers designate 10th–90th percentiles.

Lumbar punctures (LP:s) were performed on five occasions between January 2010 and May 2011, both for differential diagnostic purposes and to assess the potential impact by HIV on the cognitive impairment. CSF NFL, t-tau, p-tau, Aβ42, sAPPα and sAPPβ were analyzed with methods described elsewhere [11, 17, 18] or by the manufacturer (NF-light ELISA kit; Uman Diagnostics AB, Umeå, Sweden) and results are presented in Figure 1. The CSF biomarker profile showed a pattern with high t-tau with a mean value of 1,200 ng/L (range 1,080–1,350 ng/L) (ref <400 ng/L), high p-tau with a mean of 169 ng/L (range 153–182 ng/L) (ref <80 ng/L) and a marked decreased mean level, 212 ng/L, of Aβ42 (range 130–280 ng/L) (ref >450 ng/L). The NFL measurements were slightly higher than in HIV-negative subjects with AD, but significantly lower than in patients with HAD. The NFL values were all close to the upper normal reference level (<1,850 ng/L above 59 years), and above the age-dependent cut-off level in one of the five samples (1,890 ng/L). sAPPα and sAPPβ were, in accordance with AD, not decreased, with mean values of 970 ng/L (range 826–1,117 ng/L) and 377 ng/L (range 305–423 ng/L) respectively.

In addition, CSF was analyzed for HIV RNA, white blood cell (WBC) count, β2-microglobulin, neopterin, IgG and albumin. Serum and plasma was sampled for HIV RNA, CD4 cell count, albumin, IgG, neopterin and β2-microglobulin. At the first sampling CSF HIV RNA was elevated with 221 copies/mL, with a corresponding plasma HIV RNA level of 46 copies/mL. On the following CSF exams, CSF HIV RNA was suppressed (<50 copies/mL). Plasma HIV viral load was below 50 copies/mL on all five occasions. The immunological analyses showed a continuously intact blood–brain-barrier with a normal albumin ratio and CSF WBC count was normal (0–3 cells/uL). CSF neopterin was elevated with a mean value of 6.6 and demonstrated an increased from 4.7 to 8.4 during the time period (ref <5.8 nmol/L). CSF β2-microglobulin was slightly elevated, mean 2 (range 1–2.5 mg/L) (ref <1.8 mg/L).

## Discussion

Due to the effectiveness of ART and increased access to treatment, the HIV cohort is ageing and life-expectancy is increasing [20–22]. The prevalence of age-related co-morbidities is increasing in the HIV-infected population [23].

In this report, we present the biomarker profile in a patient with treated HIV-infection who developed cognitive decline at the age of 63 years. Her history and clinical presentation was consistent with AD, but HIV-related neurodegenerative disorders and symptomatic CSF escape [5, 6] had to be considered. Repeated LPs was performed and the biomarker profile showed an AD-pattern with markedly lowered CSF Aβ42 and elevations of t-tau and p-tau.

In AD, the characteristic histopathological findings are extracellular accumulations of amyloid β in senile plaques and intracellular neurofibrillary tangles of hyperphosphorylated tau [24–26]. The biomarker profile in CSF reflects the changed protein metabolism in AD. The decreased level of Aβ42 is believed to be a result of the aggregation into plaques, and an elevated level of CSF t-tau and p-tau caused by neuronal damage and the formation of neurofibrillary tangles of hyperphosporylated tau protein [10]. Proteolytic processing of the amyloid precursor protein (APP) by either α- or β-secretase results in the two soluble forms sAPP-α and sAPP-β which remain unaltered or mildly elevated in AD [10]. sAPP-α and sAPP-β decrease, often prominently, in HAD [11, 13]. The mechanism behind this is not known, but interestingly, sAPPs and also Aβ42 decrease significantly also in HIV-infected patients with opportunistic infections [11] and in HIV-negative subjects with other CNS infections [27, 28]. The patient in this report had normal levels of sAPPs.

NFL is the main component of large myelinated axons and a CSF biomarker of subcortical neuronal damage. As a marker of white matter disease, levels are elevated in other conditions such as vascular dementia [29], fronto-temporal dementia [30] and MS [30, 31]. In AD, NFL may be moderately elevated [29, 32], particularly in older patients where a mixed pathology of other diseases, such as vascular dementia, may be present [19] and patients with late onset AD who may demonstrate white matter pathology on radiological investigation [33]. Very high levels of NFL are normally found in patients with HAD [34, 35]. Our patient had only slightly increased CSF NFL, much lower than patients with HAD.

Neuroinflammation, demonstrated by elevations of immunological markers in CSF such as WBC, neopterin, β2 microglobulin, MCP-1, IL-10 and TNF α, among others, is well established as a consequence of CNS HIV infection. Inflammation may also persist in patients on stable ART despite viral suppression in CSF [8, 36]. Neuroinflammation also occurs in AD, however studies have mostly investigated markers of glial activation and results have been conflicting, and currently no inflammatory marker is proposed in the criteria for AD [37]. This patient showed a mild elevation in both neopterin and β2-microglobulin, which is a normal finding in patients with HIV-infection.

AD developed at an earlier age in this patient as compared to other family members and it cannot be excluded that her HIV infection, and a possible HIV CSF escape, contributed to an accelerated progression of the AD-process and neurocognitive decline. Interestingly, the levels of AD-related biomarkers in our case where at it´s extremes of the distribution for HIV-negative patients with AD. However, it is not possible to draw any general conclusions from a single case and more studies are needed to define the detailed CSF biomarker profile in HIV-infected patients with AD.

## Conclusion

It is of great importance to define differential diagnostic tools for HIV-infected patients developing non-HIV-related neurocognitive disorders such as AD. There have been no studies of the CSF profile in patients with both HIV and AD presented in the literature so far and this is, to our knowledge, the first case presented. This patient had a clinical symptomatology and PET-scan result consistent with AD, and the CSF analyses corresponded with the diagnosis and were distinctive from those found in HAD. The biomarker profile was not overtly affected by the HIV infection, and this case report suggest that the CSF biomarker profile can be used to differentiate AD from HIV-associated cognitive disorder in patients with well treated HIV-infection.

### Consent

Written informed consent was obtained from the patient for publication of this Case report and any accompanying images. A copy of the written consent is available for review by the Editor-in-Chief of this journal.

## Abbreviations

AD: Alzheimer’s disease
ART: antiretroviral treatment
CSF: cerebrospinal fluid
HAD: HIV associated dementia
HAND: HIV associated neurocognitive disorders
